# Comprehensive Insight into Colorectal Cancer Metabolites and Lipids for Human Serum: A Proof-of-Concept Study

**DOI:** 10.3390/ijms24119614

**Published:** 2023-06-01

**Authors:** Kinjal Bhatt, Titziana Orlando, Marie-Alice Meuwis, Edouard Louis, Pierre-Hugues Stefanuto, Jean-François Focant

**Affiliations:** 1 Organic and Biological Analytical Chemistry Group (OBiAChem), MolSys, University of Liège, 4000 Liège, Belgium; t.orlando@student.uliege.be (T.O.); phstefanuto@uliege.be (P.-H.S.); 2 GIGA Institute, Translational Gastroenterology and CHU de Liège, Hepato-Gastroenterology and Digestive Oncology, Quartier Hôpital, University of Liège, Avenue de l’Hôpital 13, B34-35, 4000 Liège, Belgium; marie-alice.meuwis@ulg.ac.be (M.-A.M.); edouard.louis@uliege.be (E.L.)

**Keywords:** colorectal cancer, metabolomics, lipidomics, blood, serum, chemometrics, comprehensive gas chromatography, mass spectrometry, GC×GC–TOFMS, separation science, sample preparation

## Abstract

Colorectal cancer (CRC) ranks as the third most frequently diagnosed cancer and the second leading cause of cancer-related deaths. The current endoscopic-based or stool-based diagnostic techniques are either highly invasive or lack sufficient sensitivity. Thus, there is a need for less invasive and more sensitive screening approaches. We, therefore, conducted a study on 64 human serum samples representing three different groups (adenocarcinoma, adenoma, and control) using cutting-edge GC×GC–LR/HR-TOFMS (comprehensive two-dimensional gas chromatography coupled with low/high-resolution time-of-flight mass spectrometry). We analyzed samples with two different specifically tailored sample preparation approaches for lipidomics (fatty acids) (25 μL serum) and metabolomics (50 μL serum). In-depth chemometric screening with supervised and unsupervised approaches and metabolic pathway analysis were applied to both datasets. A lipidomics study revealed that specific PUFA (ω-3) molecules are inversely associated with increased odds of CRC, while some PUFA (ω-6) analytes show a positive correlation. The metabolomics approach revealed downregulation of amino acids (alanine, glutamate, methionine, threonine, tyrosine, and valine) and myo-inositol in CRC, while 3-hydroxybutyrate levels were increased. This unique study provides comprehensive insight into molecular-level changes associated with CRC and allows for a comparison of the efficiency of two different analytical approaches for CRC screening using same serum samples and single instrumentation.

## 1. Introduction

**Figure 1:**
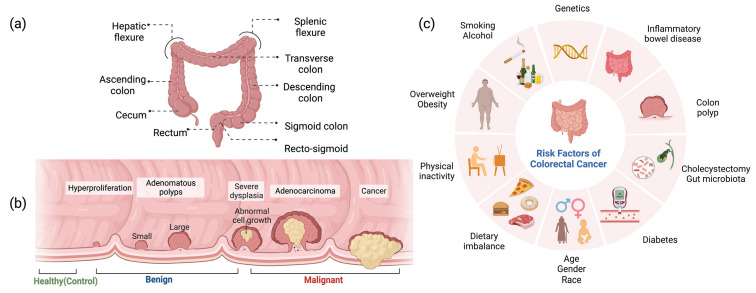
(**a**) Anatomy of colon and rectum. (**b**) Progression of colorectal polyp to colorectal cancer. (**c**) Overview of the risk factors associated with colorectal cancer development.

According to the World Health Organization (WHO), colorectal cancer (CRC) is the third most commonly diagnosed cancer and accounts for the second most cancer-related deaths globally [1]. The International Agency for Research on Cancer (IARC) projects a significant increase in the global burden of CRC, with a projected rise of 56% in the number of new cases per year between 2020 and 2040, totaling over 3 million cases annually. The estimated number of deaths from CRC is projected to increase by 69% during the same period, with approximately 1.6 million deaths worldwide expected in 2040. This increase is anticipated to be most prominent in countries with a high Human Development Index [1]. The abnormal growth of tissue protruding from epithelial cells of colorectal mucosa develops into a polyp and progresses into a tumor. If it remains benign, it is known as an adenoma or precancerous neoplastic lesion; when the tumor progresses to the carcinogenic stage, it is known as adenocarcinoma or malignant CRC tumor [2]. As shown in Figure 1, Multiple risk factors have been linked to an increased risk of developing CRC. These risk factors involve a medical history of cancer (individuals or their relatives), inflammatory bowel disease (IBD), colon polyps, cholecystectomy, and diabetes mellitus. The lifestyle-associated risk factors include physical inactivity, overweight and obesity, alcohol consumption, cigarette smoking, and inappropriate dietary patterns (a diet high in red and processed meat; a diet low in fiber, fruits, vegetables, calcium, vitamin D, and dairy products). Furthermore, age, gender, race, and gut microbiota are also considered influencing risk factors for CRC [3]. 

The current standard tests suggested by European guidelines to maintain quality assurance in the screening and diagnosis of CRC include the Guaiac Fecal Occult Blood Test (gFOBT), Immunochemical FOBT (iFOBT or FIT), flexible sigmoidoscopy (FSIG), colonoscopy, and tissue biopsy [4,5]. The endoscopy-based approaches are invasive but sensitive, while stool-based approaches are non-invasive but less sensitive. Although, these guidelines acknowledge the newer screening techniques such as computed tomography (CT) colonography, capsule endoscopy, and stool DNA testing as emerging possibilities. At the same time, they do not recommend using them for screening and diagnostic purposes for the average-risk population [5]. Therefore, there is a need for a less invasive and more sensitive analytical method for the screening of CRC.

The rapid advancement of high-throughput “omics” approaches, such as metagenomics, transcriptomics, proteomics, metabolomics, lipidomics, microbiomics, and volatolomics, offers potentially less invasive alternatives than available techniques to develop novel biomarkers for CRC screening that could contribute to its clinical management (Figure S1) [6]. Each approach offers its advantages to biomarker discovery, cancer screening, and diagnosis. The biomarker’s specificity and metabolite/lipidome identification can often fluctuate depending upon the type of biospecimens (e.g., adipose tissue, tumor tissue, dried blood spot, plasma, serum, urine, and stool) investigated [7].

Metabolomics is a comprehensive study of small molecules within biofluids, cells, tissue, or living organisms. These small molecules, commonly known as metabolites, are low-molecular-weight organic compounds usually within a mass range of 50–1500 Daltons (Da). These metabolites are typically involved as a substrate or the product of the metabolic pathway in a living organism. Collectively, a total set of metabolites and their interactions within a biological system at any specific time point is known as a metabolome. The metabolome comprises complex mixtures of thousands of molecules with a wide range of chemical diversity, which includes nucleotides, oligopeptides, amino acids, organic acids, lipids, and sugars. The analytical strategies for metabolomics are predominantly based on NMR (nuclear magnetic resonance), chromatography coupled to mass spectrometry (MS), and mass analyzer [8,9]. Various studies have reported the use of different analytical techniques such as direct infusion MS [10], GC–MS (gas chromatography–MS), LC–MS (liquid chromatography–MS) [11,12,13], UHPLC–MS (ultra-high-performance liquid chromatography–MS) [14] investigating CRC related metabolome using different biological matrix including plasma [15,16], serum [17,18,19,20,21,22], urine [23,24], and stool [25,26,27]. 

Lipidomics, or comprehensive analysis of lipids, has emerged as a new branch of metabolomics or omics cascade owing to its wide range of lipid molecules having complex hydrophobic and amphiphilic natures [28,29]. Lipids are generally classified into eight classes based on the presence of isoprene and ketoacyl groups. These are fatty acids (FAs), glycerophospholipids, glycerolipids, sterol lipids, prenol lipids, scaccharolipids, sphingolipids, and poly ketides [30,31,32]. FAs play an essential role in many biological functions as they act as fundamental components for maintaining fluidity and the structural stability of all cell membranes and also work as building blocks for making structurally more complex lipids, and as energy storage molecules [33]. Lipidomics is commonly analyzed by LC coupled with MS and MS-related techniques [34]. Several studies have explored the influence FAs in CRC using various analytical techniques. For instance, Yaping Zhang [35] analyzed serum samples using Fourier transform ion cyclotron resonance MS (FTICR MS) and GC–MS for analyzing different types of samples, including plasma [36], serum [37], and tissue [38,39].

Over the past few years, we have successfully utilized the GC×GC-TOFMS (comprehensive two-dimensional gas chromatography time-of-flight mass spectrometry) technique in various disease phenotyping and biomarker discovery applications, such as asthma, systemic sclerosis, and Crohn’s disease [9,40,41,42,43]. Our previous findings have established a strong foundation for the application of GC×GC-TOFMS in disease diagnosis, demonstrating its potential as a powerful analytical tool in the field of the omics world. The importance of dysregulated FA metabolism in cancer is well studied [31,44]. The coverage of FAs in metabolomics methods largely depends on the type of sample preparation and analytical method used, resulting in less extensive coverage of unsaturated long-chain fatty acids [C_13_ to C_21_ LFAs] and unsaturated very long-chain fatty acids [≥C_22_ VLFAs], compared to methods developed explicitly for lipids (FAs) [45]. The aim of the research is to identify sensitive and less invasive diagnostic biomarkers for CRC using GC×GC coupled with low- and high-resolution TOFMS. This study demonstrates a proof of concept for the efficacy of a less exhaustive and more automated lipidomics approach in CRC screening compared to a metabolomics workflow from an analytical perspective. Furthermore, incorporating the multi-omics metabolomics and lipidomics analytical approaches using the same sample is a powerful tool in comparing and combining the overall omics information in CRC, compared to healthy volunteers (control) and benign (adenoma) conditions, to gain a deeper molecular-level understanding of CRC.

## 2. Results

In this study, a total of 64 serum samples were analyzed, representing three different groups: adenocarcinoma (n = 20), adenoma (n = 23), and control (n = 21). The analysis was conducted using two different analytical methods, specifically targeting lipids (fatty acids) and metabolites with GC×GC-LR/HR-TOFMS. Table 1 presents a comprehensive summary of the clinical and demographic characteristics of the participants, encompassing the three distinct study groups that were subjected to analysis.

### 2.1. Lipidomic Profiling of Colorectal Cancer Serum by GC×GC-LR/HR-TOFMS

A 25 µL of serum sample was derivatized with a two-stage derivatization approach (See Materials and Methods section). In total, 40 analytes were observed. 13 saturated fatty acids (SFA), 9 monounsaturated fatty acids (MUFA), 6 PUFA polyunsaturated fatty acids (omega-3), 8 PUFA (omega-6), 1 PUFA omega-9, and 3 cholestadiene isomers. First and foremost, all the features were subjected to unsupervised analysis such as principal component analysis (PCA) and Hierarchical Clustering (heatmap and dendrogram) to observe the clustering trends (Figure S2).

The selection of CRC lipid biomarkers was based on the following three statistical criteria for multi-group analysis: First, false discovery rate (FDR) from one-way analysis of variance (ANOVA); second, the variable importance projection score (VIP score) from partial least squares—discriminant analysis (PLS-DA); third, Mean Decrease Accuracy (MDA score) from the random forest (RF) algorithm. Here, with a cut-off of FDR value < 0.05, nine significant features were identified (with *p*-value < 0.05–11 features). The variable importance in the projection (VIP) score plot is used to assess the importance of features with PLS-DA. A total of eight features were identified as significant with the cut-off of the VIP score > 1. Here, with the cut-off of MDA > 0.008 top eight features remained same in comparison to the first two tests. Higher MDA values indicate greater importance for classification accuracy. 

**Figure 2:**
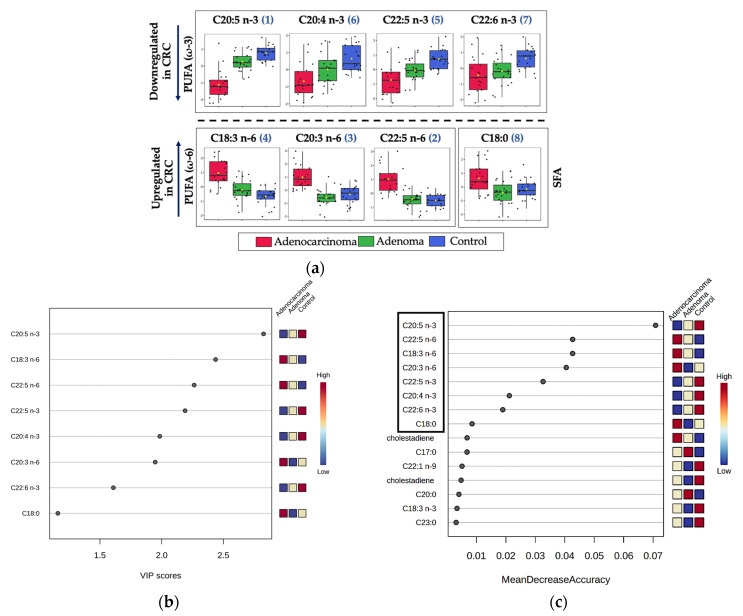
Significant features selection for lipidomics: (**a**) The comparison of the normalized concentration range of top eight significant features: ranking of features in the bracket based on its significance from one-way ANOVA (FDR value < 0.05). (**b**) VIP score plot (VIP score > 1): the colored boxes displayed on the right-hand side represent the relative concentrations of the respective analyte in each group under study (**c**) MDA feature importance plot from random forest (top eight features highlighted in black box with MDA > 0.008).

It is advantageous to use these chemometric tools in conjunction with multiple methods during the feature selection step to ensure the robustness and reliability of the selected features. This information also proves its usefulness in identifying and selecting a subset of features that achieve high classification accuracy with fewer variables. Furthermore, monitoring a small but robust set of biomarkers in a large-scale study becomes easier and more economical. As illustrated in Figure 2, the top eight features remained constant across all three tests (one-way ANOVA, PLS-DA, and RF), utilizing FDR value, the VIP score, and MDA, respectively, indicating the accuracy of these features for classification of adenocarcinoma, adenoma, and control groups based on the fatty acid profile. Out of eight features (Table 2), four belonged to PUFA (ω-3), three to PUFA (ω-6), and one to SFA. The features belonging to PUFA (ω-3) are 5,8,11,14,17—eicosapentaenoic acid, methyl ester (EPA) (C20:5 n-3); 8,11,14,17—eicosatetraenoic acid, methyl ester (C20:4 n-3); 7,10,13,16,19—docosapentaenoic acid, methyl ester (DPA) (C22:5 n-3); and 4,7,10,13,16,19—docosahexaenoic acid, methyl ester (DHA). The 3 significant features related to PUFA (ω-6) are 6,9,12 octadecatrienoic acid, methyl ester (C18:3 n-6); 8,11,14—eicosatrienoic acid, methyl ester (C20:3 n-6); 4,7,10,13,16—docosapentaenoic acid, methyl ester (C22:5 n-6). One significant feature linked to SFA is octadecanoic acid, methyl ester (C18:0).

**Figure 3:**
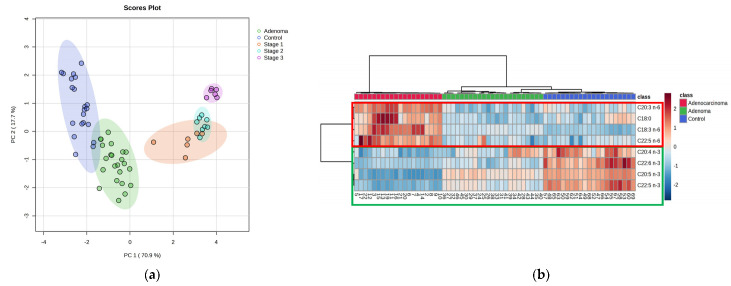
Lipidomics approach: (**a**) PCA score plot using the top eight selected features for three different groups: control (blue), adenoma (green), adenocarcinoma. Here, adenocarcinoma is divided in three stages based on pTNM staging: stage 1 (orange), stage 2 (cyan), and stage 3 (pink). (**b**) Heat map using top eight significant features selected based on feature selection criteria [control (blue), adenoma (green), and adenocarcinoma (red)].

After identifying the eight significant features, unsupervised analysis was performed. A PCA plot was generated to visualize the improvement in the clustering of the groups when compared to using all features (Figure S2a). Using the top features, a small clustering in adenocarcinoma was observed (Figure S3). Further investigation revealed that it was related to different stages of cancer based on the pTNM staging system. As depicted in Figure 3a, a clear clustering trend was observed between adenoma, control, and the different stages of adenocarcinoma (stage 1, stage 2, and stage 3). The heat map also showed a clear clustering trend between the three groups when performing with Pearson’s correlation for similarity distance measures with Ward’s linkage clustering algorithm. Thus, these findings suggest that the selected eight fatty acid analytes have a potential ability for differentiating among the adenocarcinoma, adenoma, and control groups.

**Figure 4:**
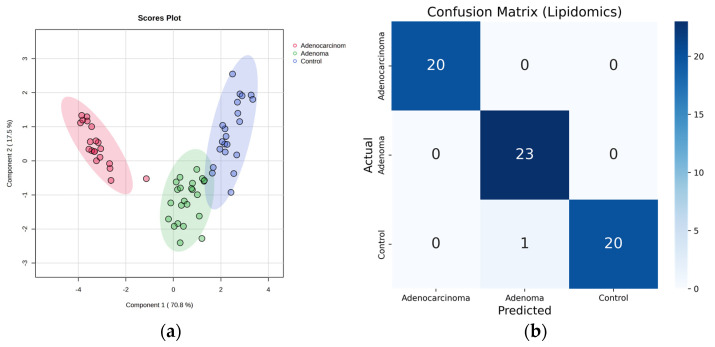
Lipidomics Approach: For top eight selected features (**a**) PLS-DA score plot (the explained % variance is shown in bracket) [control (blue), adenoma (green), and adenocarcinoma (red)]. (**b**) Confusion matrix of random forest.

To evaluate the efficiency of the selected features using a lipidomics workflow, two supervised approaches, namely PLS-DA and RF, were used (Figure 4). Upon performing a 5-fold cross-validation for PLS-DA, the accuracy, the goodness of fit (R^2^), and goodness of prediction (Q^2^) were found to be 0.93, 0.94, and 0.93, respectively, compared to using all features where the values were 0.75, 0.89, and 0.93, respectively. Here, the package has built-in support for cross-validation, eliminating the need to split the data into training and validation sets. One-third of the samples from the dataset were randomly selected as a validation set to obtain an average OOB error estimate. The model was built on the remaining two-thirds of the samples and used to predict the classes of the validation set. The misclassification rate of the validation set was calculated and repeated multiple times to obtain an average OOB error estimate. The OOB error was reduced from 0.203 (using all features) to 0.015 (using selected features). The selected features exhibited significantly low-class error rates of 0, 0, and 0.0476 for adenocarcinoma, adenoma, and control, respectively.

### 2.2. Metabolomic Profiling of Colorectal Cancer Serum by GC×GC-LR/HR-TOFMS

**Figure 5:**
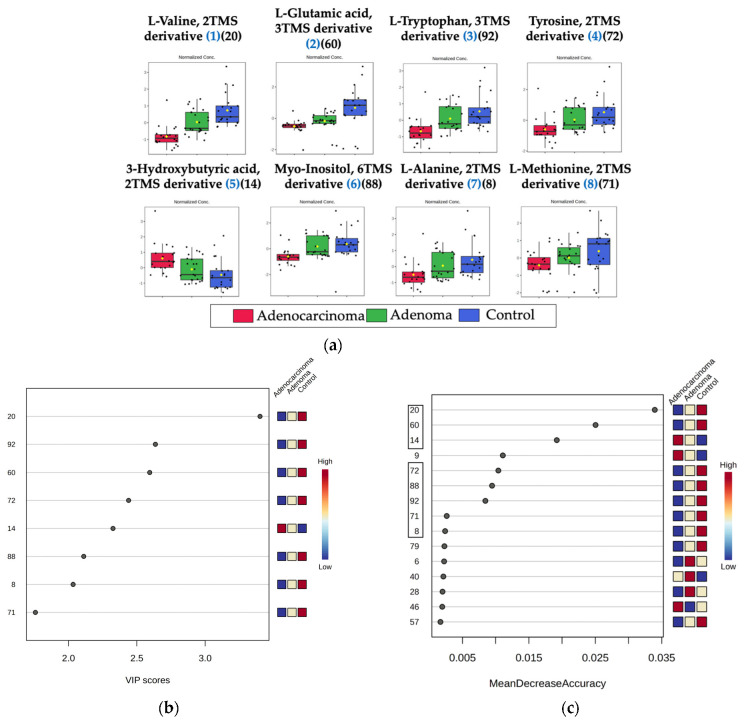
Significant features selection for metabolomics: (**a**) The comparison of the normalized concentration range of top eight significant features: ranking of features in a bracket based on its significance from one-way ANOVA (*p*-value < 0.05). (**b**) VIP score plot (VIP score > 1.75): the colored boxes displayed on the right-hand side represent the relative concentrations of the respective analyte in each group under study. (**c**) MDA feature importance plot from the random forest (features highlighted in a black box with MDA > 0.0023).

The same 64 serum samples were analyzed with a metabolomics approach. The approach involved a two-stage derivatization process in which 50 µL of serum was used for derivatization. In total, 230 analytes were observed after the removal of artifacts (i.e., solvent interference, siloxanes). Out of which, 105 metabolites were putatively identified. The metabolite’s nature ranged widely from amino acids, carboxylic acids, carbohydrates, fatty acids, nucleoside, purine, and vitamins. Firstly, all the features were analyzed using unsupervised analysis (PCA, heatmap, and dendrogram) to observe clustering trends (Figure S4). However, there was not any clear or partial separation observed among the adenocarcinoma, adenoma, and control groups. Therefore, the same set of chemometric tests used for lipidomics was applied to find significant features for CRC screening with a metabolomics approach. Upon applying a one-way ANOVA test with a *p*-value cut-off of 0.05, 11 features were identified as significant. Applying a more stringent FDR cut-off for the metabolomics approach resulted in only five features being identified as significant out of 105. Therefore, the top 11 features with a *p*-value < 0.05 were selected, which were the same as the top 11 features of the VIP score (>1.6) plot. Moreover, 13 features were found to be significant with an MDA > 2 cut-off. Out of these, the top eight features from all the tests remained common in all the tests (except for one feature, according to MDA, which was in the ninth position). Using these chemometric tools in conjunction, we identified and selected a subset of eight features (Figure 5) (Table 3) that achieved high classification accuracy in distinguishing adenocarcinoma, adenoma, and control groups (Table S1). As shown in Figure 5, out of the top eight significant metabolites, six belonged to the amino acid class. Among these, three amino acids (L-alanine, L-methionine, L-valine) were non-polar, uncharged aliphatic amino acids, and one amino acid (L-tryptophan) was a non-polar, uncharged aromatic amino acid. Additionally, one amino acid was a polar acidic amino acid, which was L-glutamic acid, and one was a polar aromatic amino acid, which was L-tyrosine. All of these amino acids were found to be downregulated in CRC. In addition, the six-carbon cyclic polyol (sugar alcohol) Myo-inositol was also downregulated in CRC [43]. On the other hand, the four-carbon organic acid 3-hydroxybutyric acid was found to be upregulated in CRC.

**Figure 6:**
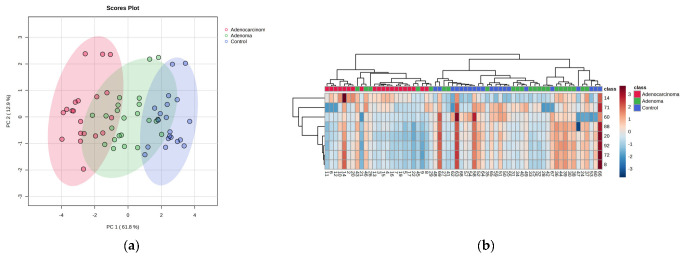
Metabolomics Approach: (**a**) PCA score plot using top eight selected features for three different groups: control (blue), adenoma (green), and adenocarcinoma (red). (**b**) Heat map using top eight significant features selected based on feature selection criteria [control (blue), adenoma (green), and adenocarcinoma (red)].

After identifying the eight significant features, unsupervised analysis was performed. A PCA plot was generated to visualize the improvement in clustering of the groups when compared to using all features (Figure S4a). However, sample number 47 from control group was found to be affecting the separation (Figure S5a,b). It is important to note that sample number 47 was not identified as an outlier when performing the Grubbs test with all features. However, it needed to be excluded to analyze the selected features’ actual separation capacity. As depicted in Figure 6a, a clustering trend with slight overlap was observed between adenoma, control, and adenocarcinoma classes. However, it was not possible to separate the different stages of adenocarcinoma based on pTNM staging using the metabolomics approach (Figure S5c). The heatmap also showed similar results. The overlap between control and adenoma group was slightly higher than that between adenoma and adenocarcinoma, which could be due to the fact that adenoma samples represent an early precancerous stage that is closer to the control group than the adenocarcinoma group.

**Figure 7:**
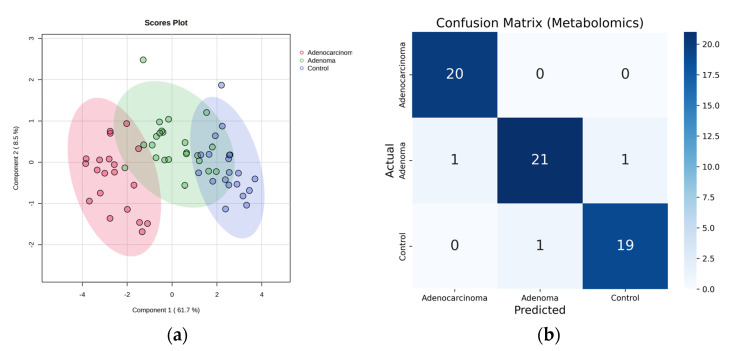
Metabolomics Approach: For top eight selected features: (**a**) PLS-DA score plot [control (blue), adenoma (green), and adenocarcinoma (red)]. (**b**) Confusion matrix of random forest.

To assess the efficiency of the selected features using a metabolomics approach, supervised analysis techniques such as PLS-DA and RF were employed (Figure 7). Upon performing a 5-fold cross-validation for PLS-DA, the accuracy, R^2^, and Q^2^ were found to be 0.81, 0.92, and 0.90, respectively, compared to using all features where the values were 0.50, 0.95, and 0.65, respectively. The out-of-bag (OOB) error was reduced from 0.375 (using all features) to 0.0476 (using selected features). The selected features exhibited significantly low-class error rates of 0, 0.087, and 0.05 for adenocarcinoma, adenoma, and control, respectively.

**Figure 8:**
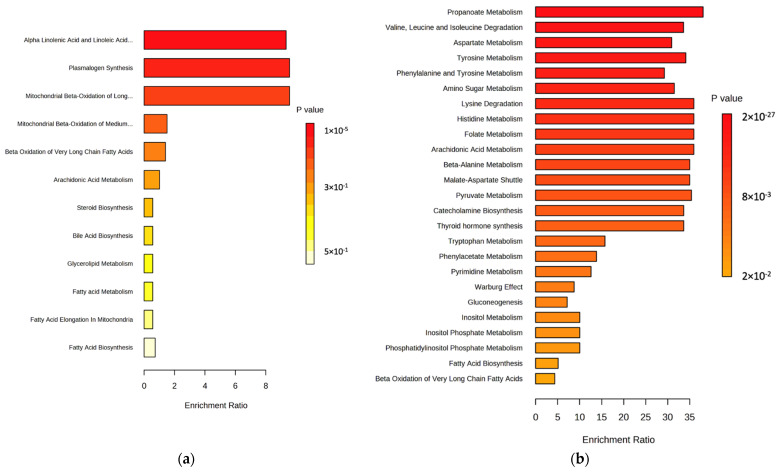
**Quantitative Enrichment Analysis:** (**a**) Lipidomics approach (adenocarcinoma and control). (**b**) Metabolomics approach (adenocarcinoma and control). (Here, the enrichment ratio is computed by observed hits/expected hits in the specific metabolic pathway).

**Figure 9:**
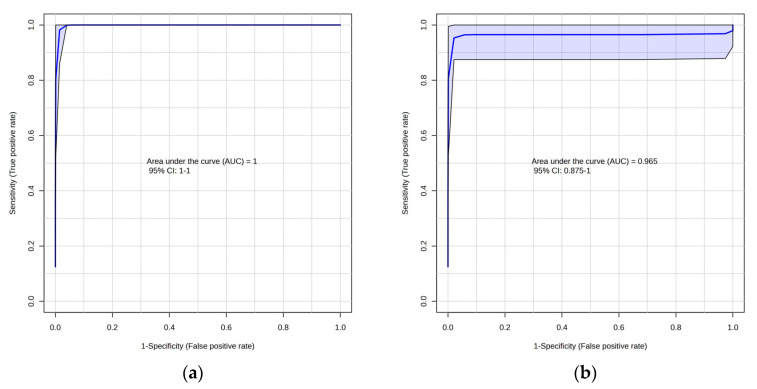
(**a**) Receiver operating characteristic (ROC) curve analysis based on PLS-DA for the lipidomics approach (adenocarcinoma and control); (**b**) ROC curve analysis based on PLS-DA for the metabolomics approach (adenocarcinoma and control).

Furthermore, all the features of the lipidomics and metabolomics approaches were subjected to QEA (Figure 8) to find the biologically meaningful groups of metabolites in data. For the lipidomics approach, the alpha linolenic acid and linoleic acid metabolism remains the key metabolic pathway (Figure 8) affected for CRC. For the metabolomics approach (Figure 8b), amino acid pathways were majorly affected. Receiver operating characteristic (ROC) curves were generated to evaluate the ability of the biomarkers to distinguish CRC during the screening process (Figure 9).

## 3. Discussion

Comprehensive lipidomics and metabolomics analytical workflows were implemented using GC×GC-TOFMS for the screening of CRC using the same sample from three different groups: adenocarcinoma, adenoma, and control/healthy volunteer. Typically, biomarker investigations for CRC screening are performed using either lipidomics or metabolomics approaches separately, which limits the ability to compare their performance using the same samples.

**Figure 10:**
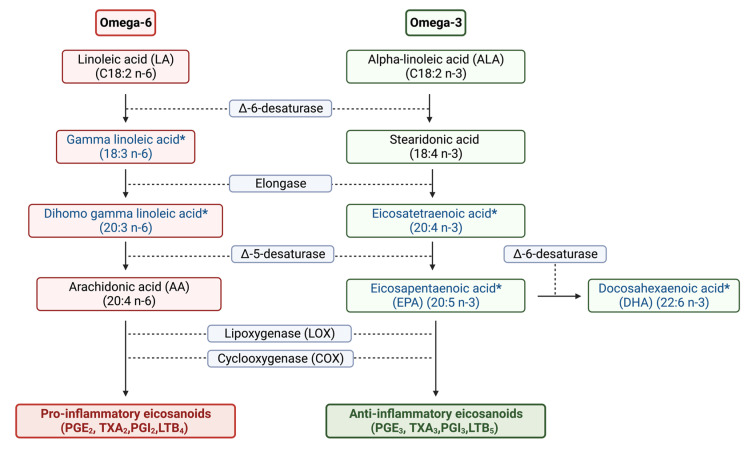
Short schematic summary of pathways for the synthesis of long-chain omega-6 and omega-3 PUFA [53]. Prostaglandin E (PGE), thromboxane A (TXA), prostacyclin (PGI), and leukotriene B (LTB) (* fatty acids were found to be significant in CRC screening using the lipidomics approach).

In this study, a comparison of results from both approaches suggests that the lipidomics approach has advantages over the metabolomics approach for CRC screening. The lipidomics approach is less time-consuming and can be automated with a dual head set autosampler accommodating two different syringe volumes for sample preparation. While comparing both approaches’ efficiency for CRC screening using same set of samples, the lipidomics approach (Figure 3) seemed to perform more efficient than metabolomics (Figure 6). Moreover, the lipidomics approach demonstrated an ability to potentially differentiate between different stages of adenocarcinoma, adenoma, and control samples while monitoring fewer analytes (Figure 3). The results of lipidomics suggest that long and very long-chain PUFA (ω-3) and PUFA (ω-6) play a major role in the progression of CRC [48], which are usually not efficiently detected using the metabolomics approach. Overall, the identified significant features having omega-6 were upregulated in CRC (adenocarcinoma > adenoma > control), while the PUFA (ω-3) were downregulated in CRC (adenocarcinoma < adenoma < control). Figure 10 illustrates that both PUFA (ω-3) (such as eicosapentaenoic acid (EPA)), and PUFA (ω-6) (such as arachidonic acid (AA)) share a common biochemical pathway mediated by the same enzymes but exert distinct physiological effects through the production of different types of prostanoids (subclass of eicosanoids). Series 2 and 4 eicosanoids are more inflammatory, while series 3 and 5 eicosanoids are less inflammatory, based on their pro-inflammatory or anti-inflammatory properties, respectively [49]. PUFA (ω-6) have been implicated in the synthesis of pro-inflammatory prostanoids, which are known to promote inflammation and have been reported to be up-regulated in CRC [49,50]. Conversely, PUFA (ω-3) has been linked with the synthesis of anti-inflammatory prostanoids, which have also been reported to be down-regulated in CRC [51,52].

A few studies have identified palmitic acid (C16:0) and stearic acid (C18:0) as significant fatty acids in CRC using metabolomics approaches [54,55,56]. However, unsaturated LFAs and VLFAs were found to be significant only using the lipidomics but not the metabolomics approach. This may be due to differences in sample preparation and the relative abundance of these fatty acids. A tailored sample preparation method for lipids can more efficiently extract unsaturated LFAs and VLFAs, which are present in lower abundance than SFAs and MUFAs in blood. Moreover, metabolomics approaches need to maintain broad selectivity towards various classes of metabolites, which can affect the extraction and detection rates of these fatty acids. Therefore, investigating the same serum sample using both approaches can reveal unique molecular changes involved in CRC. Using the metabolomics approach, the most frequently identified metabolite class perturbated in CRC is amino acid. The proteogenic amino acids (alanine (Ala), glutamic acid (Glu), methionine (Met), tryptophan (Thp), tyrosine (Thr), and valine (Val)) are downregulated in CRC. Amino acids can be preferentially metabolized to provide energy for the increased metabolism of cancer cells, act as precursors for the excessive synthesis of nucleotides in cancer cells or neutralize the heightened production of reactive oxygen species (ROS) by cancerous cells. Additionally, amino acids can also function as transcriptional or epigenetic regulators, fueling cancer-specific processes [54,57]. 3- Hydroxybutyrate, or beta-hydroxybutyrate (BHB), is a ketone body metabolite synthesized in the liver mitochondria from acetyl-CoA is a product of fatty acid degradation. It also serves as an essential energy source for the body. The level of 3- Hydroxybutyrate was increased in CRC compared to adenoma and control [54]. We acknowledge the limitation related to the limited number of samples in each cohort to make any definitive statements. However, these results serve as a proof of concept that can be used for power calculation and to guide the design of a larger-scale study. As discussed in detail in our previously published work that utilized the same sample preparation protocol [58], the importance of structural chromatographic separation using GC×GC–TOFMS for lipid analysis provides valuable insights without needing in-depth MS/MS investigations in untargeted analysis. The optimized sample preparation and known lipid molecules for CRC screening make it feasible to transfer the method to ^1^D GC–MS, which is cost-effective and scalable. This becomes highly challenging for the metabolomics approach, as it requires increased separation power of GC×GC while monitoring hundreds of metabolites.

## 4. Materials and Methods

### 4.1. Chemicals, Standards, and Samples

The derivatizing agents for the lipidomics sample preparation method, 0.5 M sodium methoxide (CH_3_ONa), and 20% boron trifluoride (BF_3_) methanolic solution, were purchased from Thermofisher and Sigma-Aldrich, respectively. N-heptane was purchased from Biosolve. A Supelco 37 FAMEs standard mixture and n-alkanes mixture (C_7_-C_30_) were purchased from Sigma-Aldrich and Millipore Sigma, respectively. The methyl undecanoic acid in heptane used as an internal standard was obtained from Reagecon, Ireland. A standard solution of 10 ppm FAMEs in dichloromethane, 10 ppm n-alkanes in hexane, and 500 ppm of methyl undecanoic acid (IS-1′) in n-heptane were prepared.

The derivatizing agents for the metabolomics sample preparation method, methoxyamine hydrochloride (MeOX), and N-methyl-N-(trimethylsilyl)- trifluoroacetamide (MSTFA) were purchased from Sigma-Aldrich. Methanol was purchased from Biosolve. The internal standards for the metabolomics approach, glycine-2,2-d2, succinic acid-2,2,3,3-d4, fumaric acid-2,3d2, and 4,4′-dibromooctafluorobiphenyl, were purchased from Sigma-Aldrich. A solution of glycine-2,2-d2 (IS-1) (100 µg/mL in milli-Q water), succinic acid-2,2,3,3-d4 (IS-2) (100 µg/mL in milli-Q water), fumaric acid-2,3-d2 (IS-3) (100 µg/mL in hexane), and 4,4′-dibromooctafluorobiphenyl (IS-4) (100 µg/mL in hexane) were prepared once a week. MeOX (30 mg/mL in pyridine) was made daily just before the derivatization step.

This study utilized pooled human plasma purchased from TCS Biosciences (Buckingham, UK) as Quality Control (QC) samples at every five injections for both methods. The certified reference standard SRM 1950 “Metabolites in frozen human plasma” was procured from NIST (National Institute of Standards and Technology, Rockville, Maryland, USA). The blood samples were collected in BD^®^ Vacutainer^®^ SST™ 3.5 mL dry tube (#cat 367957) containing an inert gel barrier that separates serum from the blood clot during centrifugation. The clotting time was 30 min at room temperature, after which tubes were centrifuged at 2000 RCF at 4 °C for 10 min. At the end, 100 µL of serum per sample was stored at −80 °C. Before the sample preparation serum sample was thawed at room temperature for 15 min. A total of 64 serum samples with both the analytical techniques (metabolomics and lipidomics) were analyzed (Table 1). 

### 4.2. The Instrumental Method

The GC×GC–TOFMS analysis was performed with a Pegasus 4D (LECO Corporation, MI, USA) equipped with Agilent 7890 GC (Santa Clara, CA, USA) coupled to Multipurpose autosampler (GERSTEL, Linthicum, MD, USA). The analysis was performed using a normal column set configuration. The first dimension (1D) column was Rxi-5Sil–MS (30 m × 0.25 mm ID × 0.25 µm df) (5% diphenyl-95%dimethylpolysiloxane phase), and second dimension (2D) column was Rxi-17Sil MS (2 m × 0.25 mm ID × 0.25 µm df) (equivalent to a 50% diphenyl-50% dimethylpolysiloxane phase). A base deactivated guard column (2 m × 0.25 mm ID) was installed before the injector. 

For the lipidomics analytical method, the starting oven temperature was 55 °C (2 min), then the temperature was increased to 155 °C at 30 °C/min, was followed by a ramp of 2 °C/min to reach till 245 °C. At last, 300 °C temperature was achieved by 30°C/min, holding it up for 2 min. The modulation period was 8 s (hot pulse: cool time between stages, 0.40: 3.60). The total run time for the GC method was 54.1 min. The modulator temperature offset was +15 °C. With positive-mode electron ionization (EI) at 70 eV, a mass range of 45–700 m/z was collected at an acquisition rate of 150 spectra/s. Transfer line and ion source temperatures were maintained at 250 °C and 230 °C, respectively.

For the metabolomics analytical method, the GC method, had a starting oven temperature of 50 °C for 2 min, followed by a ramp of 3 °C/min to reach 240 °C. Lastly, 300 °C was achieved by 30 °C/min and held for 5 min. The modulation period, modulator temperature offset, and MS method were the same as the lipidomics method (as mentioned above).

The same chromatographic and MS methods were used for GC×GC–HRTOFMS analysis performed with Pegasus GC-HRT 4D (LECO Corporation, St Joseph, MI, USA) equipped with an Agilent 7890 GC (Santa Clara, CA, USA) for the respective analyses of lipidomics and metabolomics.

### 4.3. Sample Preparation

**Figure 11:**
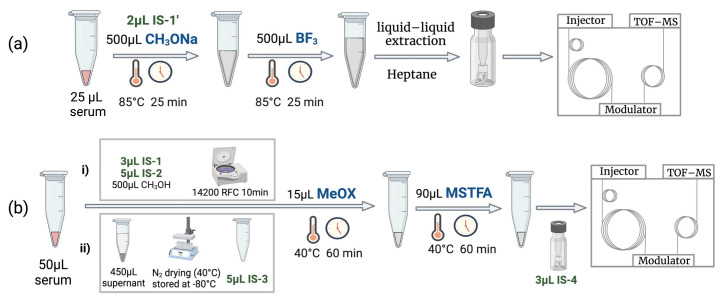
Sample preparation workflow: (**a**) lipidomics (**b**) metabolomics [(**i**) centrifugation step and (**ii**) sample drying step].

The sample for the lipidomics method was prepared as mentioned by Bhatt K. et al. [58]. Additionally, 2 µL of 500 ppm of methyl undecanoic acid in n-heptane was spiked as the internal standard (IS-1′), as shown in Figure 11a. The fundamental principle behind the metabolomics sample preparation protocol (Figure 11b) was based on [9]. However, minute changes were made to adjust to different instrument sensitivities for metabolomics sample preparation. The samples were collected at room temperature, stored at −80 °C, and thawed at room temperature for 20 min before sample preparation.

### 4.4. QAQC: The Injection Sequence

**Figure 12:**
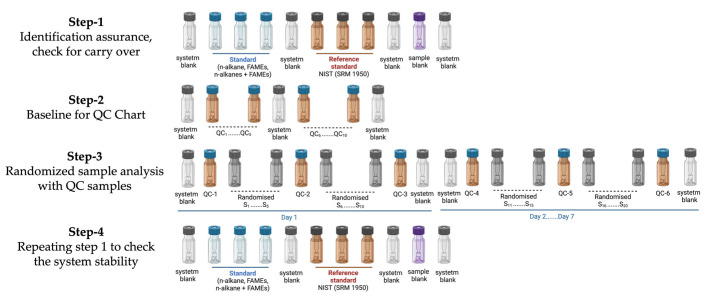
Injection sequence on GC×GC-LR-TOFMS for the lipidomics and metabolomics approaches.

The pooled human plasma was used as the QC sample at an interval of every five injections for both the methods (Figure 12). Firstly, system blank, n-alkane, 37 FAMEs standard mixture, 3 injections of NIST plasma metabolites, and sample blank were injected at the beginning and end of the entire analytical batch (before and after all samples analysis). Secondly, to create the baseline for QC Chart 10 pooled human plasma samples were injected after step 1 and before actual serum sample analysis. (Here, system blank was injected before, after 5 injections and at the end to check for carryover). All the serum samples (n = 64) were injected in a randomized order with QC samples (pooled human plasma) at an interval of every five injections. Representative analytes were selected to monitor QC samples with both approaches. For lipidomics, the average % RSD ± SD for monitored analytes of raw area, ^1^D_Rt_, ^2^D_Rt_ were 10.03 ± 1.10, 0.12 ± 0.08, and 1.40 ± 0.65, respectively (Figure S6). For metabolomics, the average % RSD ± SD for monitored analytes of raw area, ^1^D_Rt_, ^2^D_Rt_ were 17.04 ± 2.86, 0.18 ± 0.05, and 3.21 ± 3.64, respectively (Figure S7). 

Moreover, the reference standard NIST SRM 1950 (in triplicates), alkane, and FAMEs standard mixture were analyzed using GC×GC-HR-TOFMS to enhance identification confidence for both analytical approaches and to annotate MSI confidence levels [46,47]. In addition, with the metabolomics approach, actual pooled QC representing each class was also analyzed. 

### 4.5. Data Processing and Chemometrics 

The data processing step includes exporting data from ChromaTOF^®^ (ver. 4.72, LECO Corp., St. Joseph, MI, USA) with baseline correction in the Andi MS format (.cdf) and processing using GC Image^TM^ (ver. 2021r, Zoex Corp., Nebraska, USA). The putative identification of analytes is based on following parameters were used: (i) electron impact ionization mass spectra libraries (main EI MS database (mainlib), and replicate spectra database (replib)) when MS % (≥700/1000), (ii) linear retention indices (LRI) (±20 window range) (C_7_-C_30_ alkane mixture), (iii) mass accuracy (±4 ppm) (upon using HRMS detector), (iv) NIST reference standard, FAMEs standard solution, and 2D plane location. Depending upon analytes if not all at least two–three criteria were fulfilled for identification. Before applying chemometric tools, the data were pre-processed by normalizing to sample median, square root transformation, and autoscaling. The chemometric tests, including one-way ANOVA, unsupervised screening (PCA, HCA, heatmap), multi-variate supervised analysis (PLS–DA), and random forest (RF), enrichment analysis were performed using MetaboAnalyst 5.0 (Xia Lab, McGill University, Montréal, QC, Canada) [59]. The random forest machine learning algorithm was also performed in Python (version 3.9.7) using scikit-learn library (‘sklearn.ensemble.RandomForestClassifier’) which provided the same results as MetaboAnalyst. Random forest is a supervised learning algorithm that utilizes an ensemble of classification trees. At each branch, a tree is grown by random feature selection from a bootstrap sample. The prediction of the class is determined by the collective majority vote of the ensemble. RF provides useful information such as the OOB (out-of-bag) error and MDA (Mean Decrease Accuracy). The MDA is calculated by randomly permuting the values of each feature in the dataset and retraining the RF model. The difference between the baseline accuracy and the accuracy of the retrained model is calculated for each feature, representing the decrease in classification accuracy when the feature is removed from the model. The mean of the decreases in accuracy across all trees in the random forest is then calculated for each feature, and the features are ranked according to their MDA values. These values generate a feature importance plot, showing the top-ranked features in descending order of importance.

## Figures and Tables

**Table 1:** Summary of clinical and demographic characteristics of study participants.

	Adenocarcinoma	Adenoma	Control
Total no of participants (Female/male)	20 (9/11)	23 (12/11)	21 (9/12)
Female age (Mean ± SD)	66.50 ± 7.99	64.70 ± 8.69	65.81 ± 8.73
Male age (Mean ± SD)	69.45 ± 8.54	64.70 ± 8.69	65.81 ± 8.93
Location ^1^ (A/T/D/S/R/recto-sigmoid/splenic flexure)	(7/0/3/3/6/0/1)	(7/1/4/3/7/1/0)	-
pTNM staging ^2^ Stage -I (pT1N0M0 or pT1NxMx) Stage -II (pT2N0Mx or pT2NxMx) Stage -III (pT3N0Mx or ypT3N0Mx)	6 8 6	-	-
Alcohol (Yes/No)	10/10	10/13	12/9
Smoking (Yes/No)	5/15	4/19	3/18

^1^ A = ascending colon, T = transverse colon, D = descending colon, S = sigmoidal colon, and R = rectum. ^2^ pTNM—pathological evaluation of surgically resected tumor or biopsy. Here, the TNM staging system is to explain the amount and spread of cancer into patient’s body [T = size of the tumor and its spread in nearby tissue, N = cancer’s spread in nearby lymph nodes; M = Metastasis (cancer’s spread to any other part of body)]. If neoadjuvant therapy is given prior to surgical resection, the TNM category is identified by a y prefix.

**Table 2:** Putative identification and main statistical values of top eight selected features as potential biomarkers for CRC with the lipidomics approach.

Potential ID	Class	CAS	Similarity	Reverse	Probability (%)	△ LRI	(FDR) <0.05	VIP Score (>1)	MDA (>0.008)
C20:4 n-3 **	PUFA (ω-3)	132712-70-0	882	875	30.1	12	2.6 × 10^−4^	1.9847	0.0193
C20:5 n-3 *	PUFA (ω-3)	2734-47-6	890	892	58.5	8	1.1 × 10^−11^	2.8251	0.0790
C22:5 n-3 **	PUFA (ω-3)	108698-02-8	851	851	74.1	13	5.3 × 10^−3^	2.1896	0.0324
C22:6 n-3 *	PUFA (ω-3)	2566-90-7	900	910	72.9	16	2.0 × 10^−3^	1.6093	0.0193
C18:3 n-6 *	PUFA (ω-6)	16326-32-2	875	875	56.3	11	9.7 × 10^−8^	2.4366	0.0463
C20:3 n-6 *	PUFA (ω-6)	21061-10-9	919	907	69.7	12	7.5 × 10^−8^	1.9479	0.0369
C22:5 n-6 **	PUFA (ω-6)	-	897	883	28.6	18	7.5 × 10^−8^	2.2631	0.0448
C18:0 *	SFA	112-61-8	925	955	84.3	1	7.0 × 10^−3^	1.1598	0.0087

* MSI confidence level—1; ** MSI confidence level—2 [46,47].

**Table 3:** Putative identification and main statistical values of top eight selected features as potential biomarkers for CRC with the lipidomics approach.

Name	CAS	Similarity	Reverse	Probability (%)	△ LRI	*p*-Value (<0.05)	VIP Score (>1.7)	MDA (>0.0023)
L-Alanine, 2TMS derivative	27844-07-1	861	862	91.4	14	8.85 × 10^−3^	2.0337	0.0024
3-Hydroxybutyric acid, 2TMS derivative	55133-94-3	907	912	65.4	12	1.49 × 10^−3^	2.3255	0.0192
L-Valine, 2TMS derivative	7364-44-5	851	936	82.4	17	1.99 × 10^−7^	3.4019	0.0340
L-Methionine, 2TMS derivative	27844-10-6	898	898	98.7	8	3.16 × 10^−3^	1.7578	0.0026
L-Glutamic acid, 3TMS derivative	15985-07-6	878	878	94.2	13	1.90 × 10^−4^	2.5943	0.0250
L-Tyrosine, 3TMS derivative	55638-45-4	909	923	97.4	1	9.33 × 10^−4^	2.4399	0.0104
Myo-Inositol, 6TMS derivative	2582-79-8	925	925	87.7	11	3.31 × 10^−2^	2.1113	0.0095
L-Tryptophan, 3TMS derivative	55429-28-2	897	901	72.7	6	2.00 × 10^−4^	2.6359	0.0085

All the selected features have MSI confidence level—1 [46,47].

## Data Availability

Data are unavailable due to privacy or ethical restrictions.

## Supplementary Materials

The following supporting information can be downloaded at: https://www.mdpi.com/article/10.3390/ijms24119614/s1.
