# Supplementary material for: Comprehensive Insight into Colorectal Cancer Metabolites and Lipids for Human Serum: A Proof-of-Concept Study
Source: Int J Mol Sci. 2023 Jun 1;24(11):9614. doi: 10.3390/ijms24119614 (PMC10253775; doi:10.3390/ijms24119614)
Supplement: Supplementary file 1 [file ijms-24-09614-s001.zip › ijms-2406355-supplementary.pdf]

Supplementary Materials

Comprehensive Insight into Colorectal Cancer Metabolites and Lipids for Human Serum: A Proof-of-Concept Study

Kinjal Bhatt [1,*], Titziana Orlando[1], Marie-Alice Meuwis [2], Edouard Louis[2], Pierre-Hugues Stefanuto [1], and Jean-François Focant* [1]

[1] Organic and Biological Analytical Chemistry Group (OBiAChem), MolSys, University of Liège, 4000 Liège, Belgium; t.orlando@student.uliege.be (T.O.); phstefanuto@uliege.be (P.-H.S.).

[2] GIGA institute, Translational Gastroenterology and CHU de Liège, Hepato-Gastroenterology and Digestive Oncology, Quartier Hôpital, University of Liège, Avenue de l'Hôpital 13, B34-35, B-4000 Liège, Belgium; marie-alice.meuwis@ulg.ac.be (M.-A. M.); edouard.louis@uliege.be (E.L.).

* Correspondence: kinjal.bhatt@uliege.be or ikinjalbhatt@gmail.com (K.B.); jf.focant@uliege.be (J.-F.F.).

Contents

Figure S1: Overview of the omics cascade

Figure S2: Lipidomics: Unsupervised screening with all features

Figure S3: Lipidomics: PCA score plot using significant features

Figure S4: Metabolomics: Unsupervised screening with all features

Figure S5: Metabolomics: Unsupervised screening with selected features

Figure S6: Lipidomics: QC Chart

Figure S7: Metabolomics: QC Chart

Table S1. Metabolomics: A systematic feature reduction step

Omics Cascade

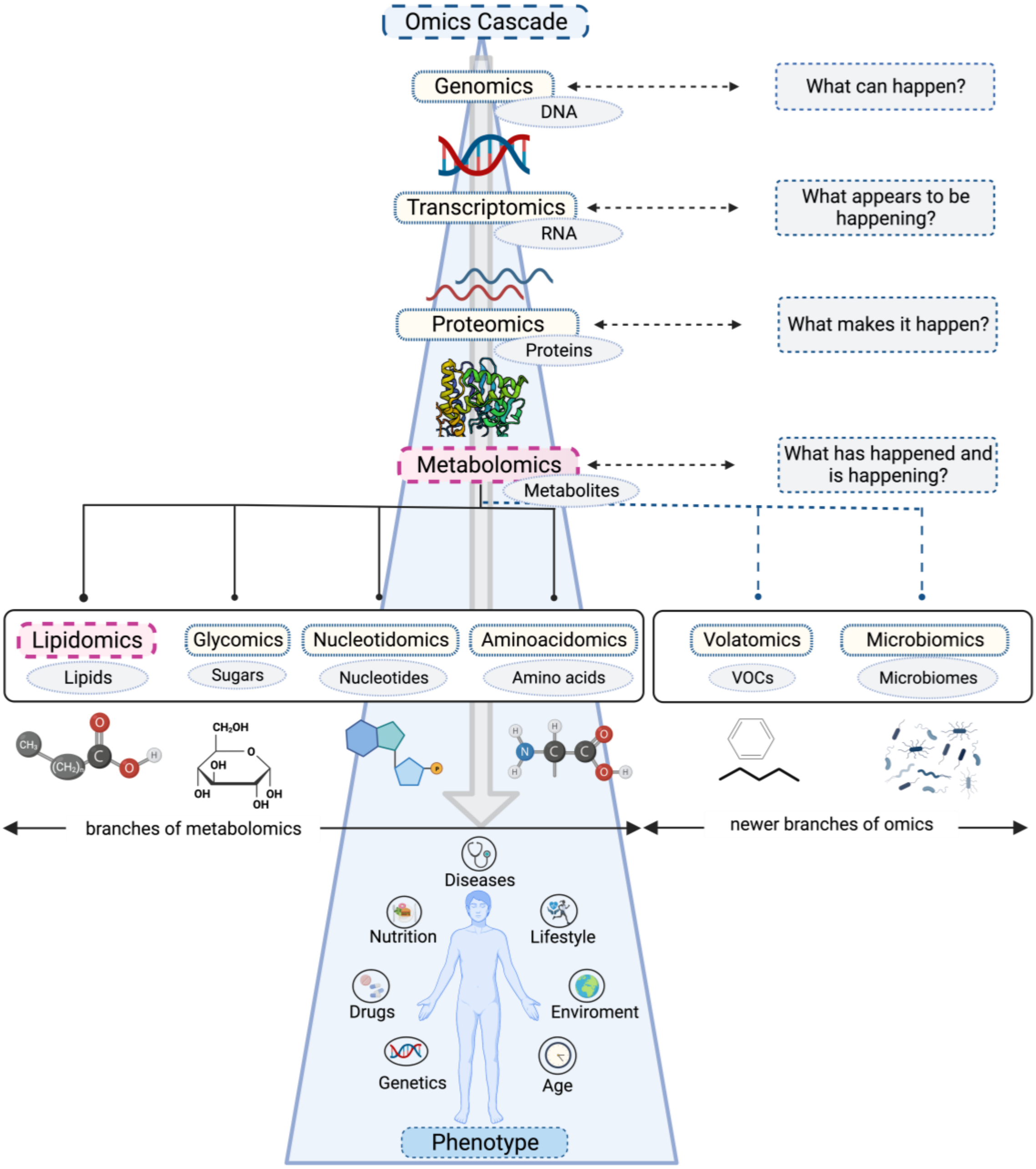

Figure S1: Overview of the omics cascade

Lipidomics: Unsupervised screening with all features

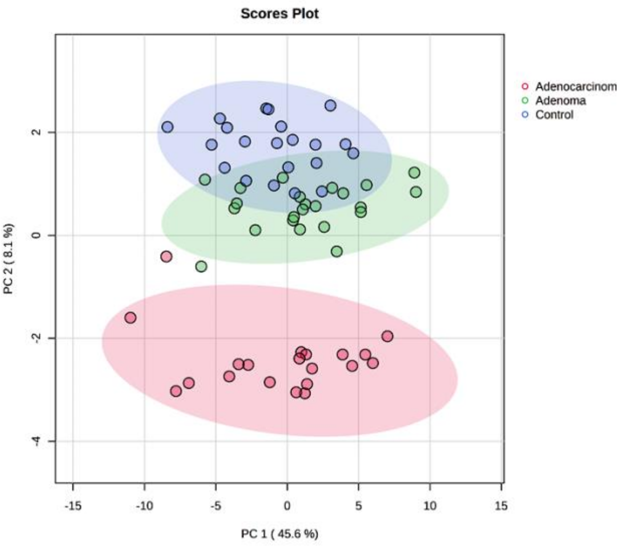

(a)

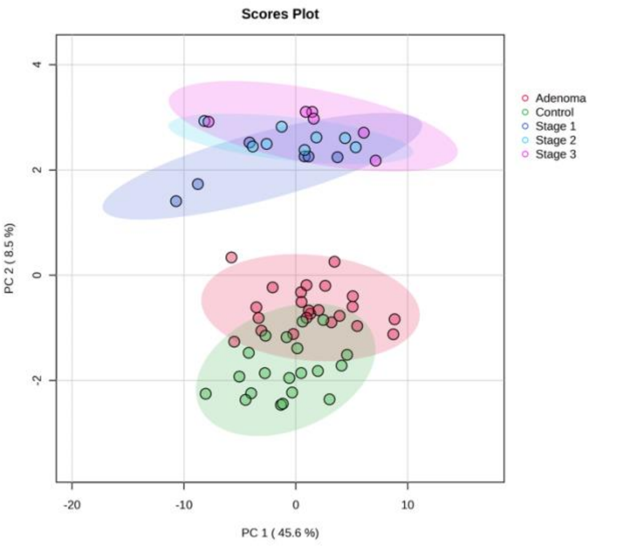

(b)

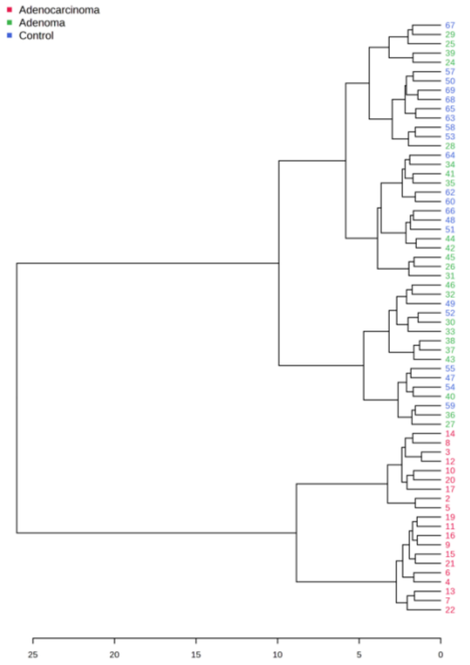

(c)

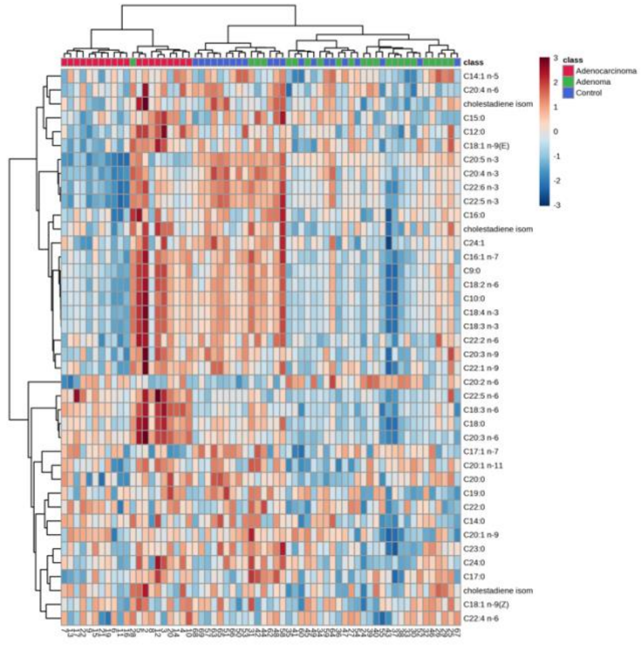

(d)

Figure S2. **Lipidomics: Unsupervised screening with all features (a)** PCA score plot for three different groups: control, adenoma, adenocarcinoma. **(b)** PCA score plot: Here, adenocarcinoma is divided in three stages based on pTNM staging: Stage 1, Stage 2, and stage 3 **(c)** Hierarchical Clustering Dendrogram **(d)** Heat map

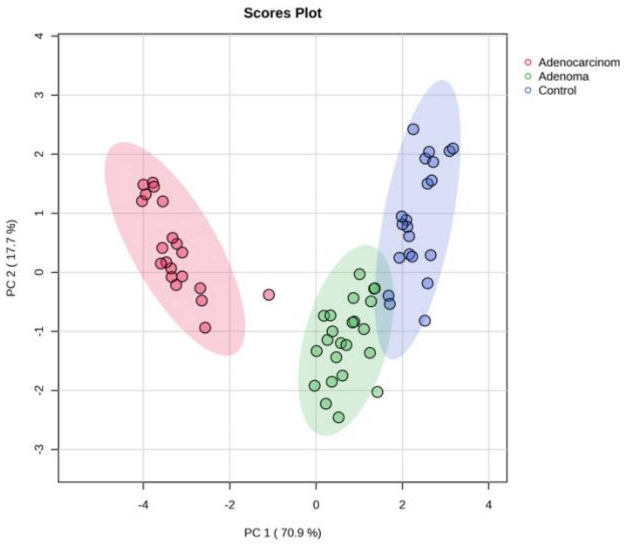

(a)

Figure S3. Lipidomics: PCA score plot using significant features (adenocarcinoma, adenoma, and control).

Metabolomics Approach: Unsupervised screening with all features

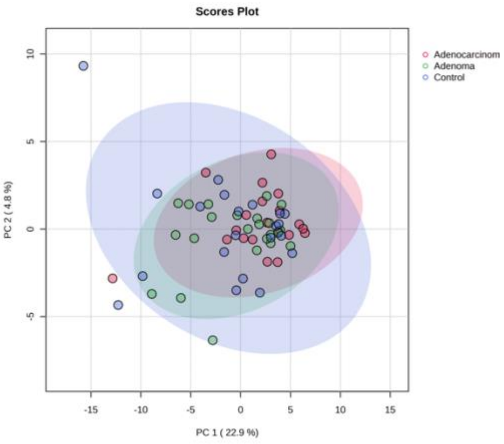

(a)

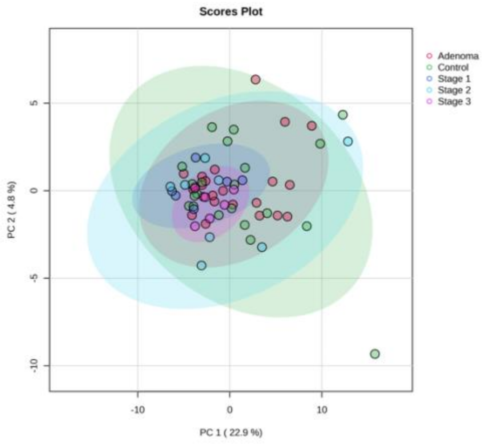

(b)

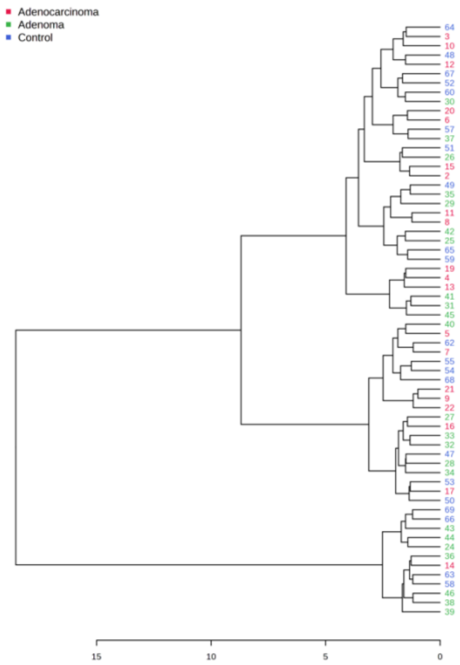

(c)

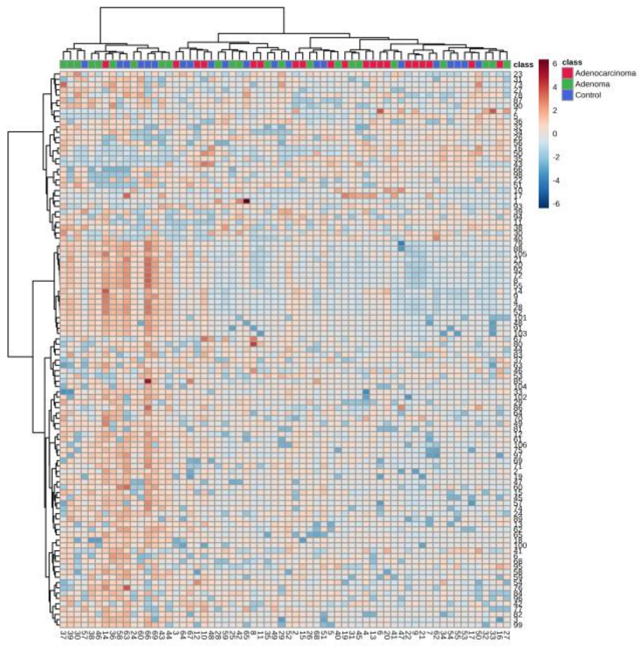

(d)

Figure S4. **Metabolomics: Unsupervised screening with all features (a)** PCA score plot for three different groups: control, adenoma, adenocarcinoma. **(b)** PCA score plot: Here, adenocarcinoma is divided in three stages based on pTNM staging: Stage 1, Stage 2, and stage 3 **(c)** Hierarchical Clustering Dendrogram **(d)** Heat map

Metabolomics Approach: significant features

Top feature	PLS-DA (Validation)			RF (1000 trees)	RF Confusion matrix (Class error)		
	Accuracy	R^2	Q^2	OOB error	Adenocarcinoma	Adenoma	Control
11	0.65	0.74	0.62	0.2500	0.250	0.261	0.238
10	0.76	0.78	0.73	0.1880	0.150	0.217	0.190
9	0.73	0.76	0.70	0.0938	0.000	0.130	0.143
8	0.79	0.81	0.75	0.0476	0.000	0.087	0.050
7	0.80	0.81	0.78	0.0476	0.000	0.087	0.050

Table S1. Metabolomics: A systematic feature reduction step [Top 11 features (feature number: 20,60,92,72,14,88,8,71,70,55,30)]

The chemometric tools were used in conjunction to identify and select a subset of features that achieves high classification accuracy in distinguishing adenocarcinoma, adenoma, and control group with fewer variables, a systematic feature reduction step was conducted. As per one way ANOVA, and VIP score top 11 metabolites (feature number (from 1 to 11): 20,60,92,72,14,88,8,71,70,55,30) were same. While according to MCA score from RF out of 11 there were 8 metabolites common with the above list. The top 11 metabolites which were common in ANOVA and VIP were again projected turn by turn by reducing one feature which was a least significant in time of p-value and VIP score at a time (as shown in Table S1).

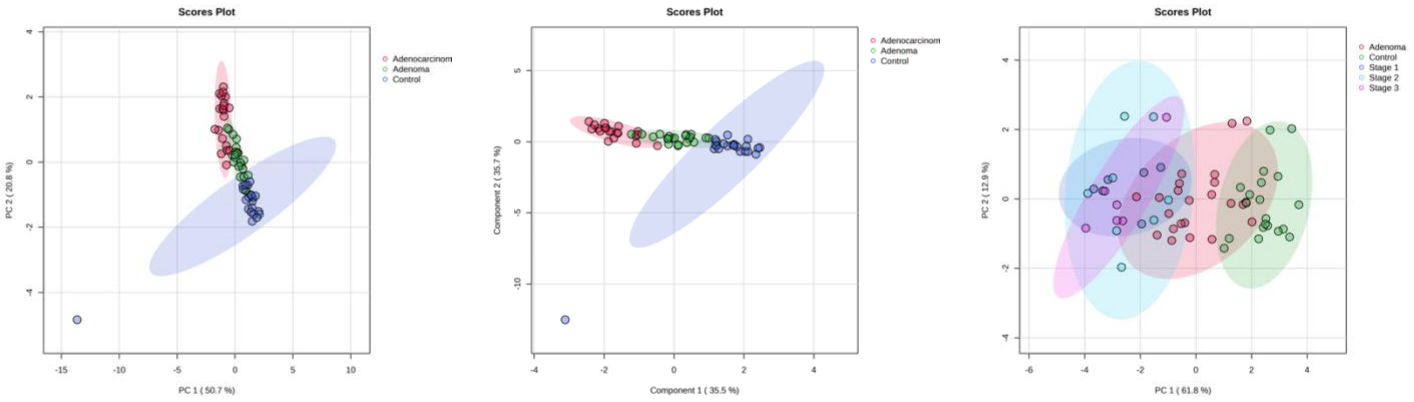

(a) (b) (c)

Figure S5. Metabolomics: Unsupervised screening with selected features (a) PCA score plot for three different groups: control, adenoma, adenocarcinoma. (control sample no 47 at bottom left corner)**(b)** PLS-DA score plot for three different groups: control, adenoma, adenocarcinoma. (control sample no 47 at bottom left corner) **(c)** PCA score plot: Here, adenocarcinoma is divided in three stages based on pTNM staging: Stage 1, Stage 2, and stage 3 (without sample no 47)

Lipidomics: QC Chart (Pooled human plasma)

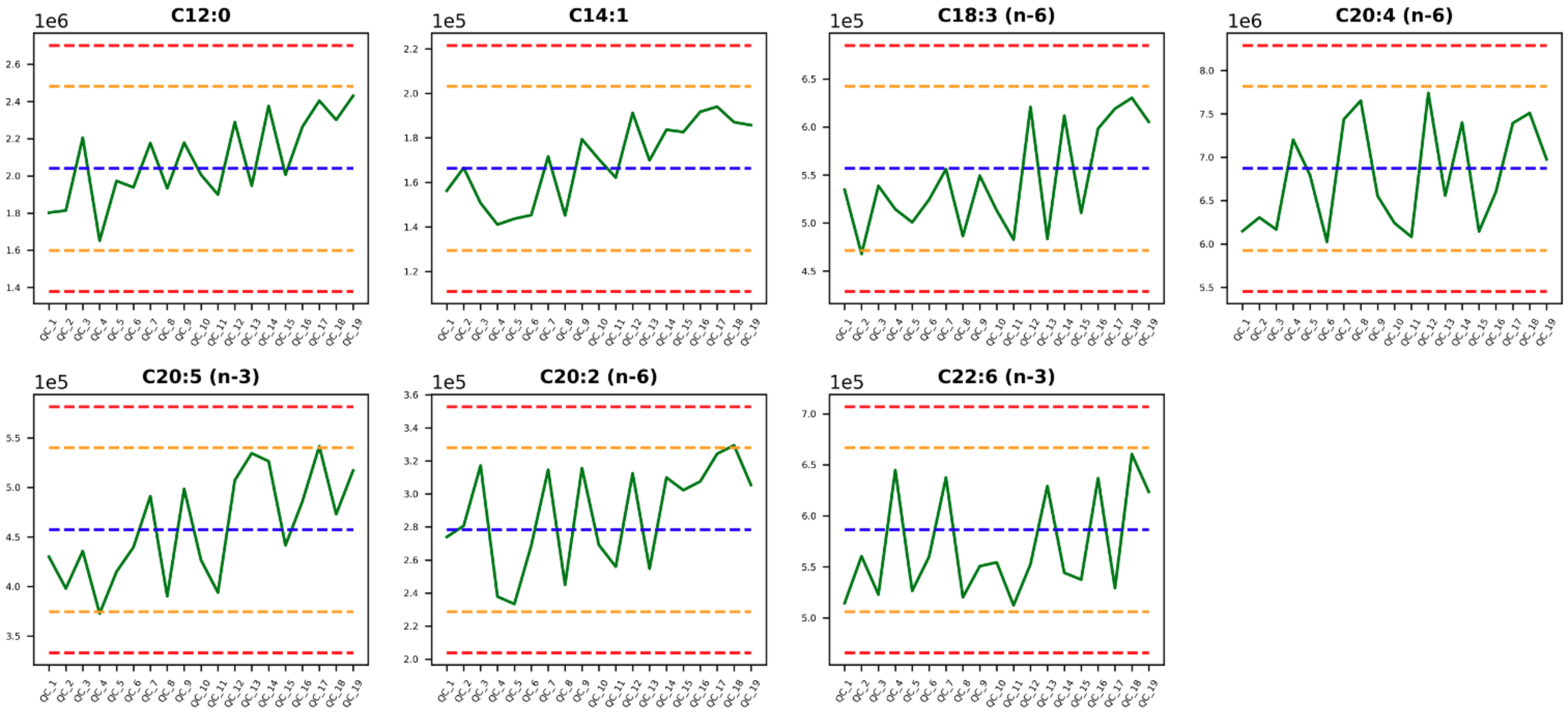

Figure S6: Lipidomics: QC Chart

Metabolomics: QC Chart (Pooled human plasma)

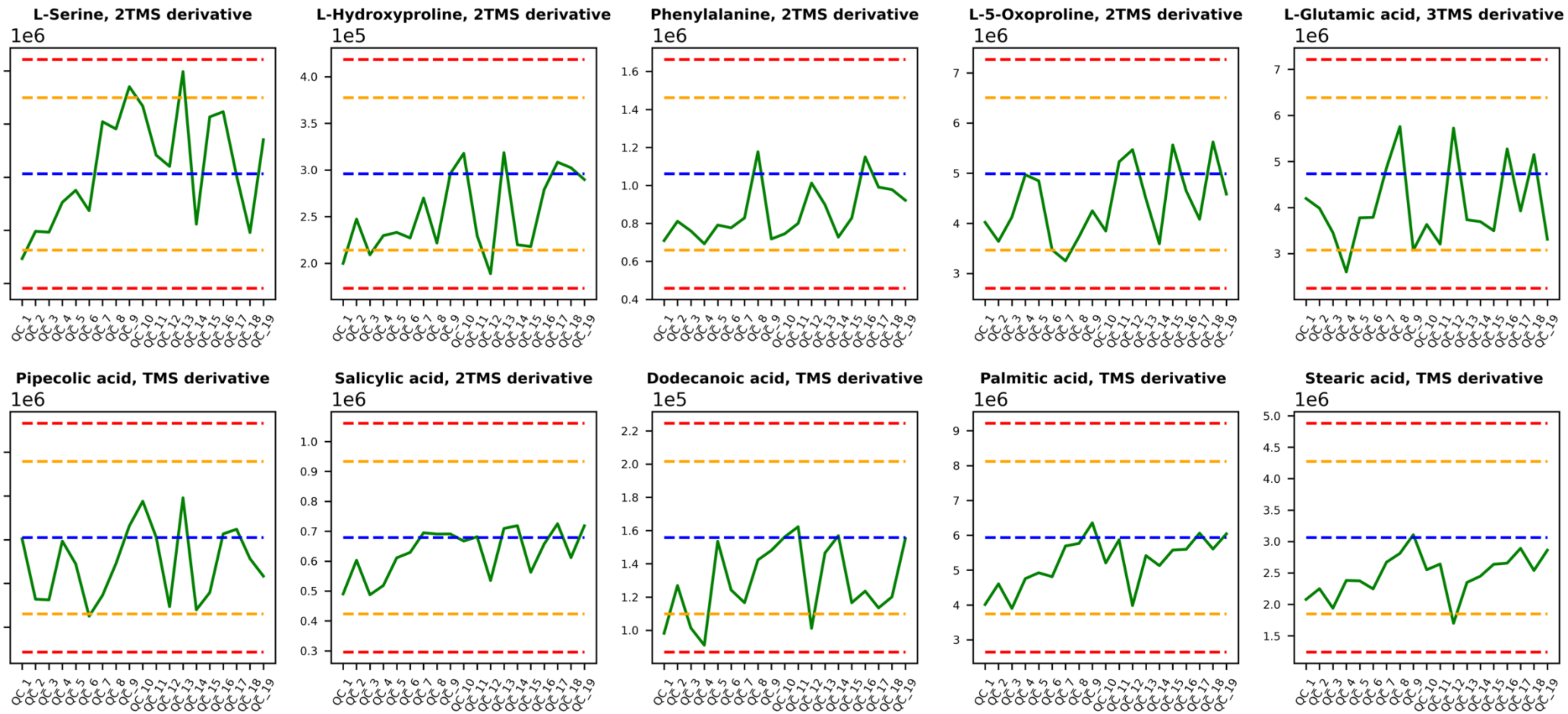

Figure S7: Metabolomics: QC Chart
